# Impact of heat stress on dairy cattle and selection strategies for thermotolerance: a review

**DOI:** 10.3389/fvets.2023.1198697

**Published:** 2023-06-20

**Authors:** Shannon L. Cartwright, Julie Schmied, Niel Karrow, Bonnie A. Mallard

**Affiliations:** ^1^Department of Pathobiology, Ontario Veterinary College, University of Guelph, Guelph, ON, Canada; ^2^Centre of Genetics of Improvement of Livestock, Animal Biosciences, University of Guelph, Guelph, ON, Canada

**Keywords:** heat stress, dairy cattle, selection, thermotolerance, physiological response

## Abstract

Climate change is a problem that causes many environmental issues that impact the productivity of livestock species. One of the major issues associated with climate change is an increase of the frequency of hot days and heat waves, which increases the risk of heat stress for livestock species. Dairy cattle have been identified as being susceptible to heat stress due to their high metabolic heat load. Studies have shown heat stress impacts several biological processes that can result in large economic consequences. When heat stress occurs, dairy cattle employ several physiological and cellular mechanisms in order to dissipate heat and protect cells from damage. These mechanisms require an increase and diversion in energy toward protection and away from other biological processes. Therefore, in turn heat stress in dairy cattle can lead numerous issues including reductions in milk production and reproduction as well as increased risk for disease and mortality. This indicates a need to select dairy cattle that would be thermotolerant. Various selection strategies to confer thermotolerance have been discussed in the literature, including selecting for reduced milk production, crossbreeding with thermotolerant breeds, selecting based on physiological traits and most recently selecting for enhanced immune response. This review discusses the various issues associated with heat stress in dairy cattle and the pros and cons to the various selection strategies that have been proposed to select for thermotolerance in dairy cattle.

## Introduction

Climate change is an issue that is currently at the forefront of many scientific, government and media discussions. The reason for this is it has the potential to result in several implications on the global environment which in turn can impact the productivity and survival of many species worldwide. The climate on the earth is determined by the amount of incoming solar energy and the ability of the earth to emit energy back to space (1). Any disruption in the balance of these processes leads to change in average temperature of the earth. It has been documented that climate change has mainly occurred due to the increase in greenhouse gasses that started in the industrial era due to increases in burning fossil fuels, deforestation, industrialization and agricultural intensification (2). Greenhouse gasses trap the energy on earth not allowing it to be emitted back to space (1). It has been shown that over the years the levels of these greenhouse gasses has continued to increase and likely will continue to increase in the future (3, 4). This increase in greenhouse gasses is due both to the rise in human activities that generate greenhouse gasses as well as to their ability to remain in the atmosphere for an extended amount of time. For example, carbon dioxide generated from the burning of fossil fuels will remain in the atmosphere for over 1,000 years (5). This increase in greenhouse gasses over the years has led to an overall warming of earth, which in turn has resulted in a number of environmental issues, which include droughts, flooding and an increase in heatwaves and hot days (1, 3).

The increase in global temperature, termed global warming, leads to several environmental issues. Due to global warming variation in precipitation has led to increased flooding in some areas and increased droughts in others (6). Additionally, reduced overall accumulation of snow has been observed, which results in depleting the water resources that are available in the summer months when it is needed most (1). Increased atmospheric temperatures have also resulted in an increase in heat waves and the number of hot days leading to a reduction in cooler days and cooling at night (1, 3). Additionally, globally heatwaves have become more frequent and intense across the majority of landmasses (3). It should however be noted these changes in climate are not the same for all parts of the world. The Intergovernmental Panel on Climate Change reports the Northern Hemisphere seems to have the greatest increases in overall warming compared to the Southern Hemisphere and warming also intensifies over land compared to sea (7). These regional difference in climate change are not associated with the level of greenhouse gasses being produced in particular areas but instead with geographical location and proximity to water (7). One example of this is Canada. On the global scale Canada produces relatively low greenhouse gas emissions and over the years has actually decreased emissions, whereas other countries have increased levels of emissions (8). However, Canada is one of the countries that has experienced the greatest warming with approximately twice the warming being observed across the country compared to the global average (3, 6). Therefore, although Canada is making efforts to reduce their greenhouse gas production, the effects of global warming will still result in environmental issues that can have negative consequences for our livestock species.

Livestock products are an important food source globally. They provide 17% of the calories consumed and 33% of the global protein consumption (9). Reports have shown that our population is continually growing indicating a need to increase production of livestock products (10). It is estimated that by 2050 our livestock production will need to increase by 70% to meet the demands globally (10). Therefore, the environmental issues associated with climate change pose a threat to the livestock industry globally. Increased levels of carbon dioxide in combination with increased ambient temperatures has led to reduced forage quality (11–13). This is because these factors change the composition of the plants making them less digestible for livestock (12). Droughts also decrease forage quality and overall yield resulting in less available good quality feed (13–15) and flooding causes poor soil quality also resulting in changes to crop quality and yields (12). This poses an issue for livestock as they may not be able to receive the nutrients, they require to sustain the mechanisms involved in production, reproduction, and overall health. Another indirect effect of global warming on the well-being of livestock species is the increased survival and distribution of vector-borne pathogens (16). Increases in ambient temperatures has allowed vector-borne pathogens to survive much longer then they typically would (16). It has also allows them to migrate and survive in other geographical locations that they typically would not have survived in the past (16). This results in increased rates of disease spread and transmission in livestock as well as the emergence of new diseases (12, 16). Global warming also impacts production, reproduction and health, of livestock species, directly as a result of heat stress (17, 18). Heat stress is a welfare and economic issue in the livestock industry, that will continue to be a problem in the future as greenhouse gasses in the atmosphere continue to increase. However, of all the livestock species heat stress seems to have a large impact on dairy cattle. Heat stress results in economic loss, with the greatest loss seen in the dairy industry (19), where about 63% of total economic losses in the United States, due to heat stress, is observed in dairy cattle versus other livestock species (see Table 1) (17). Therefore, the aim of this review is to focus on the impact of heat stress on dairy cattle and the various methods of selection that could possibly be used to breed for thermotolerance.

**Table 1:** Total economic loss by livestock species in the United States due to heat stress.

Species	Loss due to heat stress
Dairy	63.9% (about $1.5 billion/year)
Beef	15.7% (about $370 million/year)
Swine	13.4% (about $316 million/year)
Poultry	7% (about $165 million/year)

Adapted from Key et al. (17).

## Impact of heat stress on dairy cattle

Dairy cattle have been identified as one of the livestock species thar are susceptible to elevated temperatures and humidity beyond thermoneutral zones (20). Therefore, when assessing heat stress in dairy cattle, temperature humidity indexes (THI) are typically used (21). Because dairy cattle are so susceptible to heat stress several productive, reproductive and health related issues occur as a result of heat stress that has led to economic losses for dairy producers globally. Previous estimates in the United States have reported losses of approximately $1.5 billion per year in lactating cow due to heat stress (17). To understand why such losses, occur the physiological response to heat stress must first be understood.

### Physiological response to heat stress

Heat stress is defined as an environmental setting that disrupts the balance between the heat accumulation and the ability for an animal to dissipate heat (22). Dairy cattle are particularly susceptible to heat stress as milk production generates increased metabolic heat load (23). The response to heat stress in cattle can be seen through several physiological signs or symptoms as a means of trying to maintain internal homeostasis (24). When the atmospheric temperature exceeds the normal core body temperature, cattle must expend heat to maintain normal core temperatures (25). Therefore, initially, an increase in heart rate is observed (26, 27), which causes enhanced blood flow to the body surface (28–30). This allows heat from the cattle’s core to be transferred to the environment (31, 32). Additionally, evaporative mechanisms are initiated (32, 33), which include increased respiration rate and sweating (25, 34, 35). These mechanisms allow heat to be removed from the body by increasing the moisture being evaporated from these surfaces into the environment (36). In turn dairy cattle will increase their consumption of water in order to counteract water loss through evaporative mechanisms in order to avoid dehydration (37, 38). When the ambient temperature and or relative humidity levels increases beyond the level that cattle can continue to dissipate heat effectively, the core temperature of dairy cattle increases beyond normal, which is termed as hyperthermia (25, 35). During hyperthermia cattle will decrease their dry matter intake (DMI) as means of trying decrease internal heat load (25, 37). This, however, can lead to complications as feed changes have been associated with changes in microbial populations (39). It has been observed that heat stress is associated with decreased rumen pH and therefore increased risk of rumen acidosis (40, 41). This has been linked to enhanced growth of microbes that produce lactic acid in the rumen during heat stress (30). Decreased dry matter intake has also been shown to impact the villi of the gut, causing them to become shorter and fatter (42). This impacts the barrier function allowing pathogens to enter the blood stream though the intestinal lining resulting in activation of the immune response (42). This in turn leads to energy being diverted to sustaining an immune response versus going to toward growth, reproduction and production (43). Additionally, several other biological processes are shifted in order to try and maintain internal homeostasis in dairy cattle. Thyroid hormone levels decrease, as another means of trying to decrease metabolic heat load (29, 38). However, the hormones prolactin (PRL) and cortisol are increased during acute heat stress. It is thought that PRL may play a role in sweat gland function and cortisol seems to play a role in shifting immune responses (29). Conversely during chronic heat stress hormones like cortisol, growth hormone and thyroxine either decrease or remain the same (44). It is thought that the differences in hormone production during acute heat stress is due to reduced concentration of electrolytes and water as a result of losses from sweating and increased respiration rates (45). During chronic heat stress elevated levels of progesterone are observed, which may indicate a reduced conversion of progesterone to cortisol therefore resulting in reduced concentrations or no change in concentration (46).

Molecular and cellular processes may also be impacted by heat stress. Studies have shown heat stress decreases the gene expression for genes involved in transcription, RNA processing and translation and increases the expression of genes involved in heat shock transcription factor 1 and heat shock proteins (HSP), indicating a shift toward protective mechanisms (29, 47). Even the slightest increase in core temperature can cause proteins to become misfolded and can lead to the disruption of the organization of cell organelles which leads to impaired intercellular transport processes (48). This results in the loss of cellular homeostasis and leads to activation of apoptotic cascades (49, 50). Therefore, in response to the disorganization of cell organelles expression of HSP is increased (48). As internal temperature increases, and proteins become misfolded bound HSP will be released through the dissociation of heat shock factor 1 monomers from HSP (30, 51). The heat shock factor 1 monomers will then bind together forming a trimer (51). This trimer is translocated to the nucleus of the cell and binds with heat shock elements present in the promoter region of heat shock genes (52). This leads increased expression of HSP mRNA resulting in the production of inducible HSP (30, 52). Heat shock proteins can act in a couple of different ways to protect the cell during heat stress; they can facilitate re-folding of mis-folded or denatured proteins back to their native state, they prevent aggregation of mis-folded proteins and aid in the degradation of unstable proteins (25, 29, 48, 53).

### Effect of heat stress on cellular metabolism

**Figure 1:**
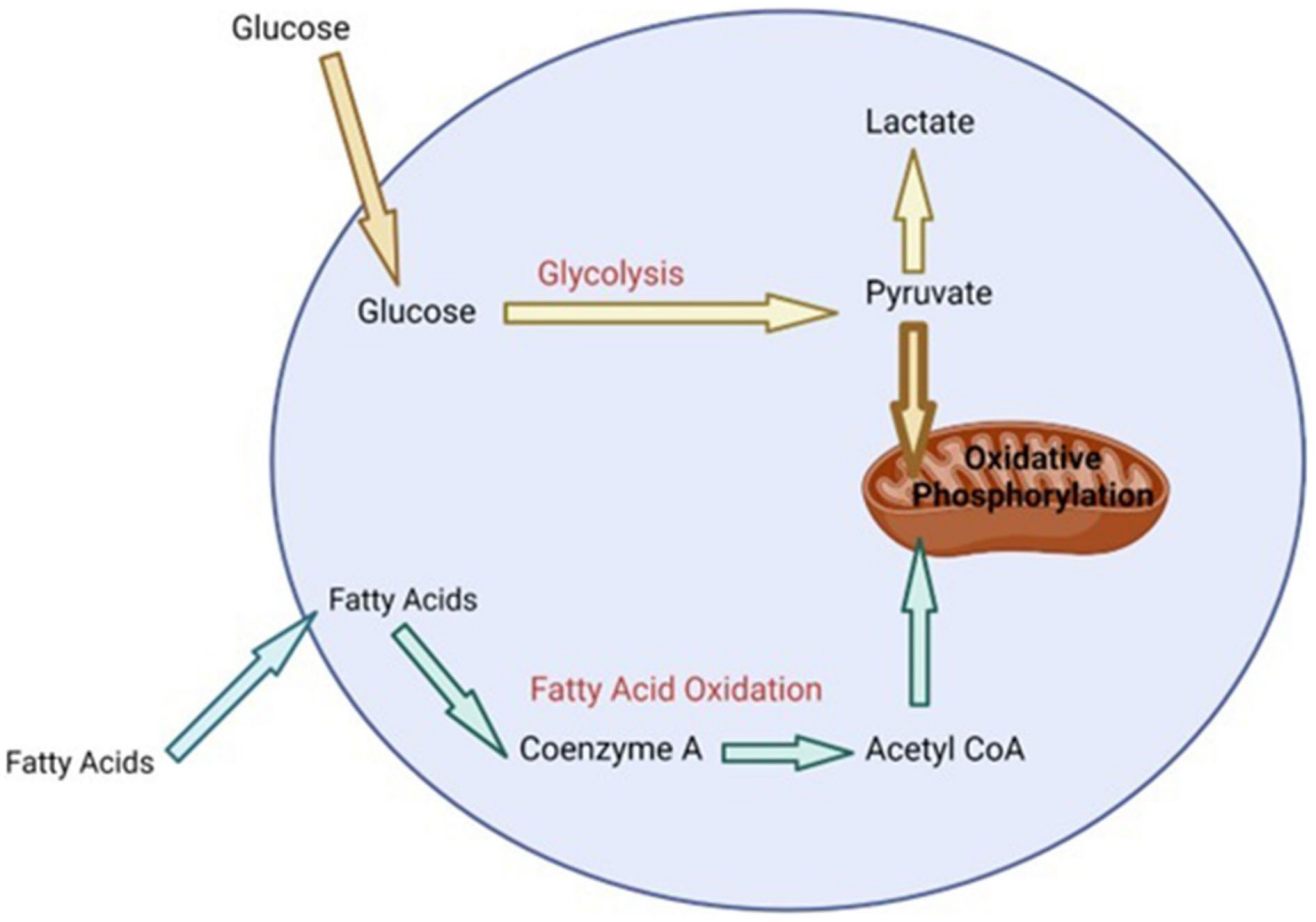
Summary of cell metabolism at homeostasis [adapted from Loftus and Finlay (55) and (64)]. Bolded text represents high levels of particular metabolic processes (oxidative phosphorylation). Bolded arrow indicates majority of the metabolite (pyruvate) transferred to mitochondria for oxidative phosphorylation. Non-bolded text and arrows represents lower concentration of metabolites and low level of metabolic processes.

**Figure 2:**
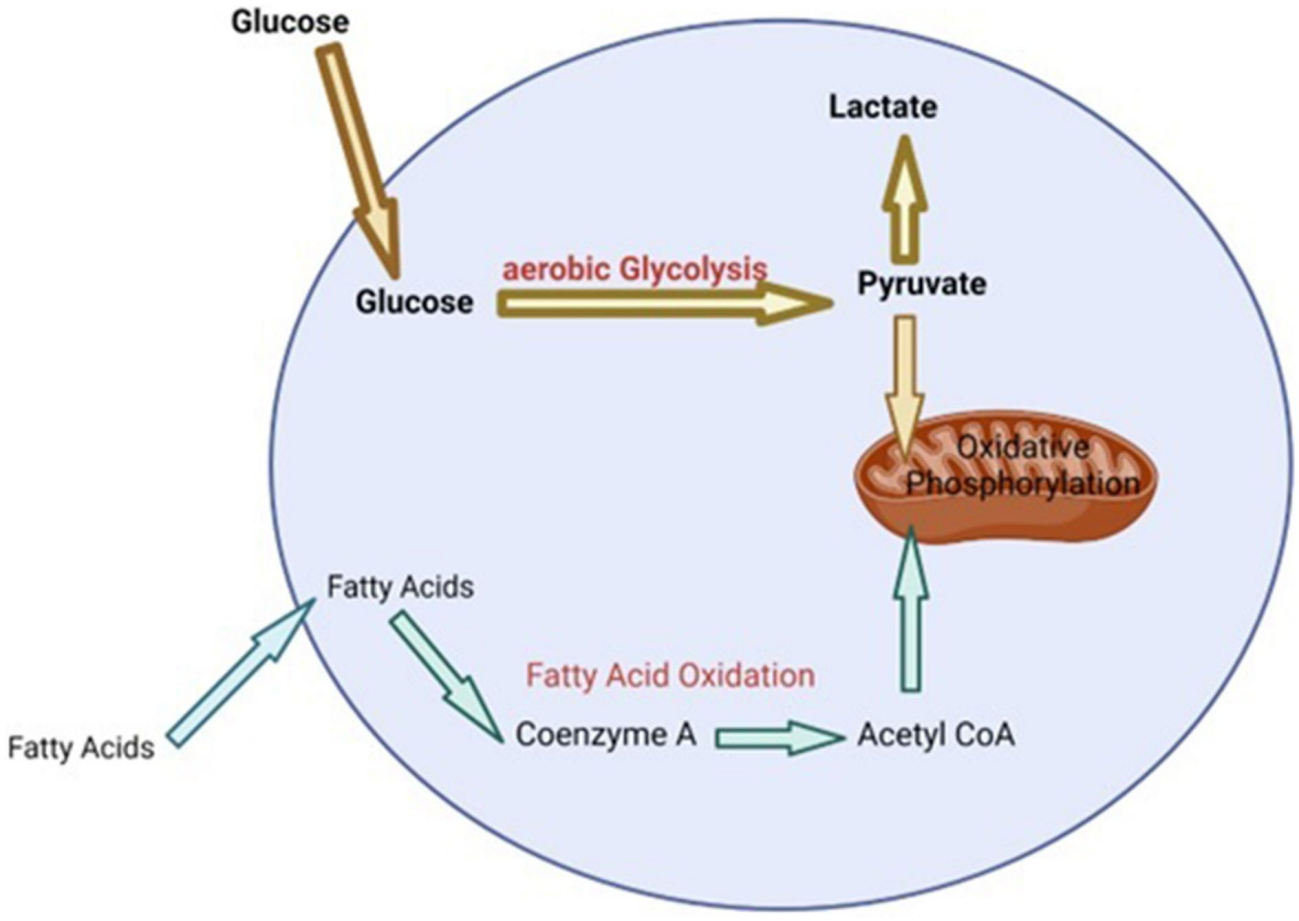
Summary of cell metabolism during heat stress [adapted from Loftus and Finlay (55) and (64)]. Bolded text represents increased concentration of metabolites or high levels of metabolic process. Bolded arrows indicate the direction a majority of the metabolites will take. Non-bolded text and arrows represent lower concentrations of metabolites or low level of metabolic process.

During heat stress there is a need to increase the bodies maintenance requirements because heat loss mechanisms require enhanced energy expenditure (54). The production of HSP, as a means of protecting cells during heat stress, also requires extra energy (55). Additionally, in dairy cattle, redistributing blood to the extremities and away from visceral organs, in order to maximize heat dissipation during heat stress, results in organ dysfunction and hypoxia of the gastrointestinal tract (56). This can lead to oxidative stress and oxidative damage of epithelial cells in the gastrointestinal tract resulting in leaky gut and the initiation of an inflammatory response (56). Metabolic pathways are the key to providing the energy and metabolites that cells require to perform various functions including initiating various effector responses (57, 58). In general, when cells of the immune system are in a homeostatic environment or anti-inflammatory state, they typically use oxidative phosphorylation that is fueled by low levels of glycolysis and fatty acid oxidation in order to provide the energy required for maintenance (55, 58, 59) (Figure 1). However, upon the initiation of an inflammatory response or when exposed to stress, cells of the immune system undergo immunometabolic reprogramming and switch to using aerobic glycolysis in order to provide the required energy to perform effector functions, as well as provide metabolites that are essential for producing molecules like cytokines, chemokines and stress proteins (55, 58, 59) (Figure 2). Studies have shown that glucose tends to be the favored energy source (40, 54) and that glycolysis is increased in dairy cattle that are suffering from heat stress (60). This suggests that dairy cattle will typically use glycolysis to supply the energy required for heat dissipation and cellular protection. Additionally, it has also been suggested that the immune system is stimulated during heat stress (60) and cells of the immune system will increase their consumption of glucose when stimulated (61). This increase in glucose consumption likely occurs in order to perform glycolysis, which is required to supply enough energy for the various physiological changes and effector functions associated with a response to heat stress.

Conflicting evidence, in dairy cattle, exists regarding the involvement of other metabolites and metabolic pathways during heat stress. It has been suggested in lactating dairy cattle that fatty acid oxidation is suppressed and reduced concentrations of circulating fatty acids during heat stress are observed (54). Its possible that lactating cattle may suppress fatty acid oxidation to reduce metabolic heat load. Whereas other studies in lactating dairy cattle have shown increased concentrations of circulating fatty acids and increased fatty acid oxidation during heat stress and it has been suggested that these metabolites and pathways may be needed to provide the extra energy required to meet the bodies demands during heat stress (62, 63). Additionally, some studies in lactating dairy cattle have shown an increase in circulation of ketone bodies during heat stress, which may be a result of reduced availability of carbohydrates for energy due to reduced feed consumption (62), whereas other studies in lactating dairy cattle have shown no effect of heat stress on the concentration of circulating ketone bodies (65).

**Figure 3:**
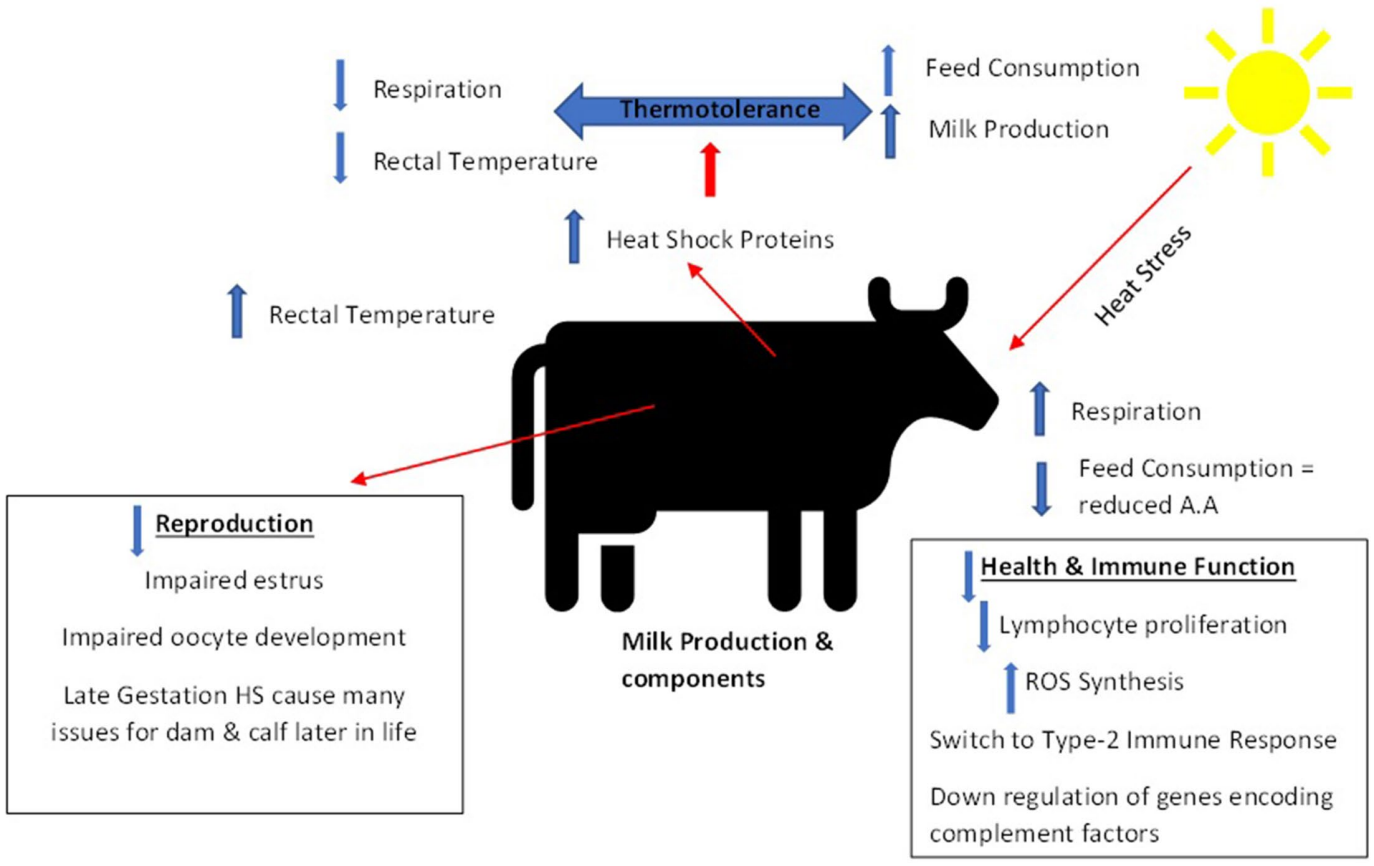
Summary of impacts of heat stress on dairy cattle [adapted from Abdelnour et al. (60)].

All the physiological and biological processes described above are important in maintaining internal homeostasis during heat stress in dairy cattle. They do however come at a cost, since energy expenditure and nutrient availability is shifted toward trying to maintain this homeostasis and away from other processes like milk production or reproduction (37). Figure 3 provides a summary of the various problems that can associated with heat stress in dairy cattle.

### Effect of heat stress on production

Some studies have suggested the THI threshold where heat stress occurs in dairy cattle is defined at the point where a decrease in milk production is observed (66). Several different THI thresholds have been reported, as a result of location or production level, that range from 60 to 78 (see Table 2). It has been shown that the genetic correlation between milk yield and THI threshold, for heat stress, is significantly negatively correlated at a value of −0.53 (66). This negative genetic correlation with milk yield is even greater as the THI increases beyond the heat stress threshold (−0.62) (66). This indicates heat stress can lead to extreme losses in milk yield, which greatly effects economics for dairy producers. Similarly, it has been shown selection for increased milk production increases dairy cattle’s susceptibility to heat stress, which is likely a result of the metabolic heat load of milk production (23). This is especially a problem in the Holstein breed, where selection in the Canadian dairy industry has put a large focus on production traits for a number of years (71). It has been reported that for each 10 kg/day increase in milk yield the heat stress threshold will decrease by 5°C (72). Similarly, during heat stress milk production in high producing dairy cattle will decrease by 0.335 kg/day versus low producing cattle will only experience a decrease of 0.158 kg/day (73). Interestingly, the same effect was also observed for heat tolerant breeds that have recently been selecting for improved production. Selecting for improved production in these breeds over the past few years has resulted in decreased tolerance to heat stress (74). Therefore, it is obvious that heat stress has an extreme negative effect on milk production, that is even more pronounced in higher yielding animals.

**Table 2:** Comparison of temperature humidity index thresholds for Holstein cattle.

THI Threshold	References
60 (Germany)	Brügemann et al. (67)
62 (Luxemburg)	Hammami et al. (68)
68 (Arizona, high producing cows over 35 kg/day)	Zimbelman et al. (21)
72 (USA)	Aguilar et al. (69)
78 (USA subtropical environment)	Dikmen and Hansen (70)

There have been several biological processes suggested for why heat stress causes such a negative impact on milk yield. One contributor to reduced milk production during heat stress is the reduction in DMI as not enough energy and nutrients will be provided to the animal to maintain normal levels of production (42). Studies have shown DMI decreases anywhere from 40 to 48% and this has resulted in milk yields decreasing anywhere from 25 to 53% relative to animals in thermoneutral environments (22, 75). However, DMI is not the only thing that contributes to reduced milk yield during heat stress. Studies have shown heat stress can both increase cell death when referring to *In vitro* studies (22) and reduce cell proliferation in mammary epithelial cells prior to calving (76). This could impact yields in the lactation following calving due to impaired mammary growth (which will be discussed further in the next section). Additionally, heat stress has been shown to upregulate genes and microRNA for protein repair and degradation and down regulate genes for cell structure, biosynthesis and transport in the mammary gland (77, 78). This indicates the mammary gland maybe putting more energy into cell survival versus milk production during heat stress.

Not only does heat stress impact overall milk yield in dairy cattle it also impacts the quality of milk. Studies have shown heat stress reduces both fat and protein yield as well as increases somatic cell count (79–82). The negative impacts of heat stress on milk components can be largely attributed to a decrease in DMI during heat stress (79). However, the reduction in protein yield may also be the result of further processes that are impacted by heat stress. As discussed previously, heat stress has been shown to alter metabolic processes, with an increased number of amino acids being found in circulation (79, 80). This could suggest that amino acids are being used to supply energy for maintenance in cattle and therefore this limits the supply that could be transported to the mammary gland for protein synthesis. These shifts in metabolism have also been shown to impact calves that experience heat stress *in utero*, which will be discussed further in the next section.

### Effect of heat stress on reproduction

Heat stress seems to affect reproduction in dairy cattle at all stages, starting from breeding through to late in gestation. These issues start right from trying to detect estrus in cattle. It has been shown that during times of heat stress 80% of estrus goes undetected, even though ovulation actually occurs (83). This is likely because heat stress reduces the signs of estrus. Heat stress causes a decrease in the production of clear stringy mucous discharge and decreases in mounting behavior, which are both common signs of estrus that dairy producers watch for (84). This would therefore make it difficult for producers to know when the optimum time to breed cattle is. This is verified by the fact that increases in THI are associated with an increased number of services to achieve pregnancy (85). Another reason why estrus may go undetected is that heat stress can cause detrimental effects to the oocyte itself. Heat stress decreases the synthesis of hormones that are crucial for estrus and pregnancy to occur. It has been shown heat stress reduces the production of luteinizing hormone and estradiol which leads to impaired follicle maturation and ovarian inactivity (86). Follicle size has been shown to be reduced by 0.1 mm for each increase in THI at estrus (84). Similarly altered progesterone levels have also been noted, which result in abnormal oocyte maturation (87). Altered hormone levels during heat stress speed up the maturation of oocytes and disrupt cytoplasmic and nuclear processes (83). This can result in several complications including issues with the fertilization process. Conception rates have been shown to be affected both before and after breeding during times of high THI, with the greatest impact occurring before breeding (88). This could be due to abnormal oocyte maturation seen during heat stress, causing impairment in the fertilization process. Similarly, it has also been noted that high ambient temperature causes increased uterine temperatures, which may be detrimental to the survival of gametes (83). This could be another factor that results in reduced conception rate during heat stress.

Despite all the complications with estrus detection and conception during heat stress dairy cattle are still able to get pregnant just not at the same rate as those being bred in cooler months. However, heat stress also leads to several complications early in pregnancy. The increased temperature of the uterine environment coupled with reduced blood flow in this area can lead to issues with the survival of the embryo (89). It can affect the embryo’s ability to attach to the uterine wall, which could lead to a loss in pregnancy (83). Heat stress can cause reduced embryonic growth and development as well as can cause a poor-quality corpus luteum, which is important to maintain pregnancy (83). All these factors would make it difficult for an embryo to survive and would lead to an increased incidence of early embryonic death during times of heat stress.

Heat stress not only effects dairy cattle early in conception or pregnancy but can also cause detrimental effects late in gestation. Dairy cattle that experience heat stress late in gestation typically experience lower milk yield in the subsequent lactation (90). This is the result of a few different issues associated with heat stress. Firstly, heat stress is associated with an increase in circulating PRL concentration (91). Prolactin is a hormone that is extremely important for mammogenesis (growth and development of the mammary gland to prepare for milk production) and lactogenesis (the beginning of milk secretion) (91). The increase in circulating PRL concentration during heat stress causes a decrease in the expression of PRL receptor genes in the mammary gland, liver and lymphocytes (91). This in turn results in reduced function and growth of the mammary gland leading to impaired lactogenesis and therefore reduced milk production in the next lactation following calving (91). The onset of lactation is also associated with an increase in cell number due to proliferation and increase in secretory capacity of each cell (91). During heat stress cell proliferation is reduced before calving, which also is another mechanism that reduces mammary growth and in turn impairs lactation performance in the subsequent lactation (91).

Late gestation heat stress not only effects the dairy cow, but also has negative impacts on the calf *in utero*. A number of studies have shown calves from dams that experiences heat stressed late in gestation have lower birth weight, weighing anywhere from 3.5 to 4.8 kg lighter then calves born to dams that were cooled (90, 92–95). This is likely due to a few different issues associated with the dam during late gestation heat stress. Firstly, during heat stress the dam will consume less dry matter in order to reduce her metabolic heat load, which would result in less nutrients being provided to the calf during the last few months of pregnancy which is the time when the most growth occurs (93). Studies have also shown the gestation length in heat stressed dams is reduced up to 4 days, which shortens the time the calf has to grow *in utero* (92, 93, 95). Placental growth and function has also been shown to be altered during heat stress (90, 93), which could be another factor that contributes to low birth weight in calves from heat stressed dams.

Heat stress *in utero* not only alters birth weight but seems to have long lasting effects on the calf in terms of overall growth, performance, and health. Heat stress *in utero* seems to shift the calf’s metabolism (90, 96). Increased levels of non-esterified fatty acid (NEFA) and beta-hydroxybutyrate (BHB) along with reduced levels of glucose were observed in calves that were heat stressed *in utero* (96). This may indicate these calves prefer to use glucose as the main an energy source vs. NEFA or BHB (96). This study also showed these calves had reduced consumption of calf starter up to weaning compared to calves from cooled dams (96). This alteration in metabolism could explain why calves exposed to heat stress *in-utero* have reduced growth up to 12 months of age (90). Not only do calves heat stressed *in utero* have reduced overall growth they also seem to have reduced reproductive performance. These calves require an increased number of services to get pregnant and on average are older when they are confirmed pregnant (90, 94). Heat stress *in utero* also results in impaired production in the offspring during first lactation. It has been observed that *in-utero* heat stress reduces milk production by 5 kg/day up to 35 weeks into the first lactation and this occurs in spite of the fact that no difference is body weight or body condition score is observed at calving between *in utero* heat stressed offspring and non-heat stressed offspring (90, 94, 97). *In utero* heat stress impacts survival, with more stillborn calves being born when the dam is heat stressed as well as a reduced number of *in utero* heat stressed calves surviving to first lactation (94). This could be a result of reduced immune function as a result of lower Immunoglobulin (Ig) G absorption (95), which will be addressed further in the next section.

### Effect of heat stress on health and immune response

Along with productive and reproductive issues, heat stress also has a huge impact on the health of dairy cattle. Several studies have indicated cattle have increased disease occurrence during times of heat stress. Heat stress can alter the rumen function and in combination with reduced feed consumption increases cattle’s risk for metabolic disorders (98). Similarly, diseases like mastitis have also been shown to have increased occurrence during heat stress (98), which could be a function of increased survival of pathogens during these times or impaired immune response. Higher incidence of mortality has also been reported, with mortality rates increasing 1.27 times during a heat wave compared the control period in dairy cattle in Southern Ontario (99). These health-related issues associated with heat stress could be due to several factors, but one of the biggest factors may be the effect heat stress has on the immune response.

Both the innate and adaptive immune system can be impacted by heat stress. Heat stress effects the adaptive immune response by disrupting the balance between T-Helper 1 (TH-1) and T-Helper 2 (TH-2) responses, and causing a responses to shift toward TH-2 (100). Additionally, cortisol is one of the main glucocorticoids produced in response to heat stress. Cortisol binds DNA and causes inhibited expression of genes that are involved in the activation of TH-1 cells and pro-inflammatory cytokine production (101). This causes the impairment of the cell-mediated immune response (CMIR), which is the response primarily responsible for the defense against intracellular pathogens. Similarly, heat stress has also been shown to play a role in down-regulating pro-inflammatory or TH-1 cytokines and the up-regulating TH-2 or regulatory cytokines (100, 101). Together, all these studies suggest a shift toward a TH-2 response or a suppressive immune response, which can significantly increase the risk to diseases caused by intra-cellular pathogens in dairy cattle.

Heat stress in dairy cattle has also been shown to lead to reduced proliferation of lymphocytes (101, 102). Lymphocytes, which include B and T-cells are activated upon pathogen exposure resulting in rapid proliferation in order to fight infection. Therefore, if lymphocyte proliferation is reduced it makes it extremely difficult for cattle to defend against pathogens. Similarly, heats stress can also cause impaired neutrophil function (103). Impaired cellular function could be attributed to a few things. Firstly it could be a result of cell damage or cell death as heat stress can increase reactive oxygen species production and reduce antioxidant defense therefore leading to cell damage due to oxidative stress (60). Addict heat stress down regulates the expression of L-selectin on the surface of neutrophils, which results in neutrophils failing to move into the site of infection (104).

Complement activation is another element of the immune system that can be impacted by heat stress. Heat stress cause the downregulation of the genes that encode various components of the complement system, as well as factor B and H (105). Complement aid in the enhancement of the antibody response and phagocytic cell function as well as damages the membranes of infected cells in order to clear pathogens. Therefore, if this system is impaired it makes it difficult for cattle to clear invading pathogens.

Heat stress can also negatively impact the immune system a during late gestation in dairy cattle. As we mentioned previously, heat stress during late gestation causes an increase in the concentration of circulating PRL, which results in decreased expression of PRL receptors. The negative feedback loop as a result of increased circulating PRL is also associated with reduced lymphocyte proliferation (106). Additionally, during late gestation, cells from heat stressed cows, display reduced oxidative burst and phagocytosis relative to cooled cows, indicating heat stress causes reductions in uptake and killing of pathogens resulting in increased susceptibility to invading pathogens (106). Furthermore, reduced specific IgG response is also observed as a result of heat stress during late gestation, compared to cooled cows (106), which indicates animals may have impaired response extra-cellular pathogens as well. Evidently, heat stress seems to cause additional strain to an already challenged immune system in pregnant dairy cattle, causing enhanced risk to various diseases.

Dairy calves are born with little to no immunity and therefore rely on immunoglobulin (Ig) from colostrum and the ability to transfer these Ig from the colostrum through the gut to provide them with immunity until their own immune system matures (107). Failure of passive transfer (FPT) indicates calves were unable to transfer an adequate amount of Ig from colostrum before gut closure and this leaves them susceptible to any pathogens they may encounter (108). Heat stress during late gestation can effect the immune system of the *in utero* calf. Calves that are born from heat stressed dams have been shown to have increased FPT (109). This FPT could be due to lower IgG in the heat stressed dam’s colostrum. However, studies have shown even when these *in utero* heat stressed calves are fed colostrum with high concentrations of IgG, they still present lower levels of IgG in their serum (93). Therefore, FPT may also be due to reduced absorption in the gut of the calf. Calves born to heat stress dams also have diminished lymphocyte proliferation, which also impacts their ability to produce and immune response and therefore their health and survival (90, 93). Considering the evidence for how heat stress negatively impacts the immune system, it is not surprising that dairy cattle and calves have increased incidence of disease and mortality during times of heat stress.

Therefore, considering all the negative effects associated with heat stress (Figure 3) and the current and future environmental challenges associated with climate change this indicates a need to identify and select animals that are more resilient to these environmental stressors.

## Selection strategies for thermotolerance

Several selection strategies for thermotolerance have been proposed as possible solutions to heat stress. These will now be discussed in the following sections.

### Selecting for reduced milk production

There have been numerous studies in dairy cattle showing various THI thresholds with which cattle will experience heat stress (21, 67, 68, 70). However, it is generally well accepted that higher producing cattle have lower THI thresholds versus lower producing cattle (21). This is likely due to the fact that milk production results in the generation of metabolic heat with increased production resulting in greater metabolic heat loads (23). Therefore, one selection strategy that has been proposed to achieve thermotolerance is to select for reduced milk production. This has been studied in Australia, where genomic breeding values related to production traits were established as indicators of heat tolerance (110). It has been shown that these traits are moderately heritable and result in a slight increase in production traits when evaluating the response to selection (110). Additionally, it was shown when selecting cows based on this selection index for heat tolerance and then exposing them to a heat challenge, the heat tolerant cows had less of a decline in yield, lower respiration rates and reduced increases in rectal temperatures versus heat susceptible cows (75). Additionally, heat tolerant cows returned to baseline yield values faster than that of the heat susceptible cows (75). Genomic breeding values based on these studies have since been released as a means of selecting heat tolerant dairy cattle (111, 112).

Although selecting for lower milk production does seem promising as a means of improving heat tolerance, it does have its limitations. One of the biggest issues with this selection index is cattle will now be bred to have reduced production traits. In fact, it was shown the genomic breeding values for heat tolerance are negatively correlated with the Australian breeding index, especially with respect to production traits at a correlation of −0.85 (110). Therefore, although heat tolerant cattle will have less reductions in yield during times of heat stress, their overall 305-day milk yield would likely be less compared to that of heat susceptible animals. Hence, in countries with varying seasons throughout the year this might not be the ideal selection strategy to establish thermotolerance in dairy cattle. Additionally, when genome wide association studies were preformed on heat tolerant animals, based on this index, no genes associated with production traits or heat tolerance were found (23). Hence, since production traits are an indicator trait for heat tolerance and not directly associated it would be worth evaluating if heat tolerance is achieved overtime when breeding using this index. Therefore, although using production traits as an indicator of heat tolerance does seem to provide some benefits during times of heat stress and it is easy to obtain large data sets on these traits, it may not be the best overall selection strategy based on the issues discussed.

### Crossbreeding and gene editing

Another potential selection strategy for thermotolerance that has been discussed is crossbreeding *Bos indicus* breeds with *Bos taurus* breeds. *Bos indicus* breeds, which are found in more tropical regions, have been shown to have a greater ability to adapt to thermal stress compared to *Bos taurus* breeds (113). Genes associated with thermotolerance have been identified in the genome of *Bos indicus* breeds (114). It has also been shown these breeds have a greater ability to control body temperature, have reduced metabolic rates and an increased ability to dissipate heat (114, 115). Additionally, it has been shown these breeds adapt well to changes in feed supply (116). Therefore, crossbreeding *Bos taurus* breeds with *Bos indicus* breeds could be a relatively easy way to introduce heat tolerance genes into *Bos taurus* breeds ultimately resulting in thermotolerance. However, it should be noted that there is evidence of thermotolerance in *Bos taurus* breeds from tropical regions (117).

There are however also some issues with using crossbreeding as a selection strategy for thermotolerance. Although, *Bos indicus* cattle possess several traits associated with thermotolerance it has also been shown these breeds typically have lower milk production (118). Therefore, by crossbreeding with *Bos indicus* breeds even though it may result in producing thermotolerant offspring these offspring will also likely have lower yields relative to *Bos taurus* breeds. Additionally, crossbreeding with *Bos indicus* breeds may result in offspring that are less tolerant to colder temperatures (118). Hence, in countries with varying seasons, such as Canada, this may not be the ideal solution, as when winter occurs it is possible these crossbred offspring would have an increased risk for cold stress.

Another genetic strategy that has been suggested is to use gene editing to introduce specific genes or mutations that result in resilience to heat stress. One example is the SLICK haplotype. This haplotype occurs due to a deletion in an exon of the PRL receptor and was first reported in the Senepol breed (119). The SLICK mutation causes changes to the coat of cattle resulting in shorter hair and lower follicle density across the coat (120). Additionally, it has been suggested sweating ability may also be increased in cattle carrying the SLICK mutation (120). Recent studies have evaluated introducing the SLICK mutation into the genome of Holstein cows (121, 122). Results from these studies have found Holstein cows with the SLICK haplotype have an enhanced capacity to regulate body temperature as well as maintain production levels during heat stress (121, 122). Therefore, introducing the SLICK mutation into *Bos taurus* breeds might be a good solution to generating thermotolerance in these breeds while maintaining production levels. The issue with introducing this mutation into *Bos taurus* breeds is that since it changes the cattle’s coat phenotype to produce a coat that allows for enhanced heat dissipation it is possible it will also in turn increase the risk of cold stress in these animals (118). Consequently, similar to what was discussed with respect to crossbreeding with *Bos indicus* breeds, introducing the SLICK mutation into *Bos taurus* breeds in countries where seasons vary might not be the most ideal solution for thermotolerance.

### Selection for physiological and cellular traits

One promising solution that could work to confer thermotolerance in dairy cattle from various locations across the world is to select for the various physiological traits that are involved in cooling during heat stress. As previously mentioned, one of the first physiological signs of heat stress in dairy cattle is increased respiration rate (36). Additionally, body temperature may also increase if cooling mechanisms are ineffective at dissipating enough heat (35). Respiration rate and rectal temperature are both traits that have been discussed to be included in selection programs to confer thermotolerance (30). Studies in dairy cattle have shown heritability estimates for rectal temperature ranging from 0.06 to 0.17 (123, 124). These estimates are in the low to moderate range indicating genetic gains are possible when selecting for this trait. There have however been relatively few studies in dairy cattle reporting estimates of heritability for respiration rate. One study did report a heritability estimate of 0.04 for respiration rate, which is in the low range indicating it would take longer to make genetic gains when selecting for this trait (124). Although genetic gains in thermotolerance may be slow when selecting for reduced respiration rate and rectal temperature, one of the benefits of this approach is milk production is not impacted. Therefore, when selecting for these traits it is possible thermotolerance can be achieved without reducing overall milk production (118).

Additionally, cellular traits have also been identified as being associated with thermotolerance. Nitric oxide synthesis is one cellular trait that has been identified as being associated with thermotolerance (38). Nitric oxide is an important molecule in facilitating the vasodilation of skin during heat stress, which helps in dissipating heat to the environment (98, 125). Therefore, by selecting for enhanced nitric oxide production it is possible vasodilation of the skin would be improved during heat stress allowing for better heat dissipation. Another avenue of investigation for selection for thermotolerance is the examination of HSP. These proteins are not only involved in the initiation of immune responses but also have a key role in response to heat stress (60). As previously mentioned, HSP are involved in the protection and repair of cells during heat stress (126). To date there have been no studies that have reported heritability estimates for HSP in dairy cattle and therefore it is difficult to know how quickly genetic gains would be made if selecting for this trait. It is however a trait that many studies identify as being associated with thermotolerance or heat stress resilience in cattle and therefore is still worth discussing as a possible trait for selection (60, 102, 118). Additionally, studies have shown that increased expression of HSP is linked to reductions in respiration rate and rectal temperature (60). Hence, by including these physiological and cellular traits in a selection index both of these traits could be improved to confer overall thermotolerance while minimally affecting milk production and avoiding a situation that results in an animal that is less resilient to cold stress.

Selecting for physiological and cellular traits seems like a promising strategy to confer thermotolerance with having little or no effect of milk production. The one issue with using these traits in a selection index is they are costly and labor intensive to measure and therefore obtaining large data sets on these traits is quite difficult (30, 127). This could be one reason why very few studies have reported heritability estimates for physiological traits and why currently no heritability estimates exist for cellular traits. Therefore, including these traits in a selection index as a means of selecting for thermotolerance is likely not feasible currently and likely will not be until less costly and labor-intensive methods can be identified for collecting large data sets on these traits.

### Selection for high immune response

Another trait that has been identified as being associated with thermotolerance is immune response (23). The ability to select dairy cattle that can mount a high immune response has been developed over many years. The idea of this started between the 1970s and 1980s where researchers showed it was possible to identify mice that had enhanced antibody response to a specific antigen (128, 129). After nine generations of selection, these mice were shown to have titers that were 30-fold higher then mice identified as low responders (128). The high antibody responding mice also had enhanced response to a wide variety of antigens as well as greater response against extra-cellular pathogens (130). This concept of selecting for high antibody response was later extended to poultry species, like chickens (131). Around this time the concept of selecting for both antibody mediated immune response (AMIR) and CMIR was also being evaluated in pigs (132). It was shown that selecting for both AMIR and CMIR simultaneously caused genetic improvement in both traits and led to pigs with overall enhanced immune response (133). Later it was demonstrated that pigs bred for overall high immune response had a balance between type 1 and type 2 responses, whereas pigs selectively bred for low immune response tended have a bias toward a type 1 immune response (134). These results suggest that breeding for high immune response would result in animals that are able to respond effectively to both extracellular and intracellular pathogens and therefore exhibit broad based disease resistance.

The method that was previously used in pigs was later adapted to measure immune responses in dairy cattle. Similar to what was done in mice, initially it was demonstrated that dairy cattle could be identified as high, average or low antibody responders (135). Later research was done to include the evaluation of CMIR as well, and showed the ability to identify both high AMIR and CMIR dairy cattle (136). A number of heritability estimates have been made throughout the years for immune response in dairy cattle (Table 3), but the most recent pedigree based heritability estimates for Holstein cattle are reported at 0.45 and 0.18 for AMIR and CMIR, respectively (139). These heritability estimates are moderate to high indicating the ability for the genes encoding these immune response phenotypes to be passed onto the next generation. Studies have also indicated the ability to use genomic selection to improve immune response. It has been shown that significant single-nucleotide polymorphisms (SNP) have been identified for both AMIR and CMIR (139, 140). These SNPs have functional properties that are associated with immune response and disease (139, 140). Combining both pedigree based information on immune response with genomic information improves accuracy in selection while also allowing quicker gains to be made when selecting for high immune response in dairy cattle (141).

**Table 3:** Heritability estimates of immune response.

AMIR	Heritability CMIR	Type
0.32–0.64	N/A	Pedigree (135)
0.25–0.42	0.19–0.49	Pedigree (137)
0.16–0.41	0.19	Pedigree (138)
0.45	0.18	Pedigree (139)
0.37	0.16	Genomic (139)

Recent evidence has shown dairy cattle identified as high immune responders may be more thermotolerant than those identified as average and low. Multiple studies have shown high immune responding dairy cattle have lower respiration rate at higher THI values compared to average and low responding dairy cattle (142, 143). Additionally, one study evaluating physiological responses to heat stress in a tie-stall facility also indicated in general high immune responders have lower rectal temperature, during a natural heat stress challenge, compared to low immune responders (143). Studies evaluating the function of blood mononuclear cells during both *in vitro* and *in vivo* heat challenges have shown dairy cattle identified as high immune responders have enhanced production of HSP70 after multiple heat challenges compared to dairy cattle identified as average or low responders (142, 144). In this same *in vitro* study it was also shown that high immune responders have a tendency to produce more nitric oxide over multiple heat challenges compared to average and low immune responders (144). As discussed above all of these physiological and cellular traits have been identified as being associated with thermotolerance and have been discussed as possible traits to select for thermotolerance. However, also as mentioned previously these traits are also costly and labor intensive to measure. Selecting for high immune response in dairy cattle is now relatively easy and cost effective and with the recent evidence of this trait also being associated with thermotolerance this may be an ideal selection strategy (145). Previous studies have also shown no difference in milk production between high immune responders and their herd mates (146). Therefore, selecting for high immune response in dairy cattle seems to be ideal cost-effective selection strategy to confer thermotolerance while maintaining production and minimizing cold stress.

## Conclusion

In conclusion heat stress is a serious issue for dairy cattle that will continue to persist as long as climate change continues to be an issue. Heat stress has resulted in large economic losses in the dairy industry due to reductions in milk production, reproductive issues and increases in treatment costs due to increased disease occurrence. This indicates a need to select dairy cattle for thermotolerance. Several selection strategies have been proposed that seem to provide the potential for breeding thermotolerant cattle, however some limitations have also been identified. Selecting for reduced milk production, crossbreeding with thermotolerant breeds and gene editing to introduce the SLICK gene all seem to be promising strategies for dairy cattle that live in more tropical environments. However, these strategies may not be ideal for cattle living in countries with seasonal variation as they could result in overall reduced milk production and may inadvertently create cattle that are more susceptible to colder temperatures. Selecting for physiological and cellular traits is a promising strategy to introduce thermotolerance while minimizing production losses and issues with cold stress. Unfortunately, this data is very costly and labor intensive to collect and therefore it is difficult to obtain large data sets on these traits. Nevertheless, recently is has been shown dairy cattle identified as high immune responders seem to be more thermotolerant compared to their herd mates. Therefore, selecting for high immune response may be an ideal solution to conferring thermotolerance in dairy cattle living in both tropical and temperate countries.

## Author contributions

SC prepared the initial review. JS, BM and NK contributed equally to providing intellectual support and editing and review. BM held the grant for financial support. All authors approved the review for publication.

## Funding

This work was supported by the Canadian First Research Excellence Fund with the grant being held by BM.

## Conflict of interest

The authors declare that the research was conducted in the absence of any commercial or financial relationships that could be construed as a potential conflict of interest.

## Publisher’s note

All claims expressed in this article are solely those of the authors and do not necessarily represent those of their affiliated organizations, or those of the publisher, the editors and the reviewers. Any product that may be evaluated in this article, or claim that may be made by its manufacturer, is not guaranteed or endorsed by the publisher.
